# Earlobe arterialized capillary blood gas analysis in the intensive care unit: a pilot study

**DOI:** 10.1186/2110-5820-4-11

**Published:** 2014-04-14

**Authors:** Sergi Vaquer, Jordi Masip, Gisela Gili, Gemma Gomà, Joan Carles Oliva, Alexandre Frechette, Simon Evetts, Thais Russomano, Antonio Artigas

**Affiliations:** 1 Critical Care Center, Sabadell Hospital, CIBER Enfermedades Respiratorias, Parc Tauli University Institute, Parc Taulí 1, 08208 Sabadell, Spain; 2 Wyle GbmH, Albin Koebis Strasse 4, Cologne, Germany; 3 Centro de Microgravidade/FENG-PUCRS, Av. Ipiranga, 6681 - Partenon, 90619-900 Porto Alegre, (RS), Brazil

**Keywords:** Acute respiratory failure, Arterialized, Capillary blood, Emergency medicine, Intensive care medicine, Mechanical ventilation

## Abstract

**Background:**

Earlobe arterialized capillary blood gas analysis can be used to estimate arterial gas content and may be suitable for diagnosis and management of critically ill patients. However, its utility and applicability in the ICU setting remains unexplored.

**Methods:**

A prospective observational validation study was designed to evaluate this technique in a cohort of mechanically ventilated adult critically ill patients admitted to a polyvalent ICU. Precision and agreement between capillary gas measures and arterial references was examined. Acute Respiratory Distress Syndrome (ARDS) diagnosis capabilities with the proposed technique were also evaluated. Finally, factors associated with sampling failure were explored.

**Results:**

Fifty-five patients were included into this study. Precision of capillary samples was high (Coefficient of Variation PO_2_ = 9.8%, PCO_2_ = 7.7%, pH = 0.3%). PO_2_ measures showed insufficient agreement levels (Concordance Correlation Coefficient = 0.45; bias = 12 mmHg; percentage of error = 19.3%), whereas better agreement was observed for PCO_2_ and pH (Concordance Correlation Coefficient = 0.94 and 0.93 respectively; depreciable bias; percentage of error 11.4% and 0.5% respectively). The sensitivity and specificity for diagnosing ARDS were 100% and 92.3% using capillary gasometric measures. Sampling was unsuccessful in 43.6% of cases due to insufficient blood flow. Age > 65 years was independently associated with failure (odds ratio = 1.6), however hemodynamic failure and norepinephrine treatment were also influencing factors.

**Conclusions:**

Earlobe capillary blood gas analysis is precise and can be useful for detecting extreme gasometrical values. Diagnosis of ARDS can be done accurately using capillary measurements. Although this technique may be insufficient for precise management of patients in the ICU, it has the potential for important benefits in the acute phase of various critical conditions and in other critical care arenas, such as in emergency medicine, advanced medical transport and pre-hospital critical care.

## Background

The use of arterialized capillary blood from the earlobe (EL) to estimate arterial blood gas content was first described by Drs. Lilienthal and Riley [1]. This technique is based on the hypothesis that blood from dilated capillaries of the EL contains a higher proportion of arterial than venous blood and thus is a good estimator of arterial gas content. Sampling from the EL has several advantages compared to arterial puncture such as reduced complication rate, minimized invasiveness and better patient tolerance. However, studies in healthy subjects and patients have shown contradictory results on the accuracy of this technique, which has limited its implementation in current clinical practice [2-8]. As suggested in a recent meta-analysis [9], arterial gasometrical values could be estimated from EL capillary samples and may be useful for clinical practice. However, limited precision was reported, which was attributed to arterio-venous capillary gas differences and a lack of standardized sampling procedures.

Rapid and reliable gasometric analytical capabilities are of prime importance in critically ill patients and can be determinants of diagnosis and treatment, such as in the onset of Acute Respiratory Distress Syndrome (ARDS). EL arterialized capillary blood gas analysis has the potential for important benefits for critical care medicine in any of its multiple arenas (ICU, advanced medical transport, emergency medicine). However, limited information exists on the utilization of this technique in critically ill patients.

We present a prospective observational validation study to ascertain whether EL arterialized capillary blood gas analysis, when collected with dedicated equipment and procedures, can accurately estimate arterial gasometrical values in critically ill patients and evaluate, directly at point of care, its capability to diagnose ARDS when used in conjunction with established clinical and radiologic criteria.

## Methods

### Subjects

Mechanically ventilated critically ill adult patients, with an indwelling arterial line and admitted to the ICU for either medical or surgical conditions, were eligible for this study. Severe coagulopathy (prothrombin time > 2.5 ratio, platelet count < 10,000 units/dl) and inability to obtain informed consent by the patient or next of kin were exclusion criteria. Sample size estimation for comparing capillary versus arterial samples was based on the method described by Walter and Shoukri [10,11]. In order to compare the two methods using the Concordance Correlation Coefficient (CCC) approach, a minimum of 30 valid data points where needed. Due to an unexpected high sampling failure ratio, 55 patients were included in a non-consecutive fashion in order to obtain 31 valid samples. Two interim analyses were performed after the inclusion of the 15th and 45th patients in order to monitor sampling success, and to adjust patient inclusion for the observed data acquisition rate and the required number of valid samples. The study protocol was in accordance with the amended Declaration of Helsinki and was approved by an Independent Ethics Committee (IRB: 2009593. Comité Ètic d’Investigació Clínica, Corporació Sanitària Universitària Parc Taulí. Sabadell, Spain).

### Procedures

Capillary sampling procedure was based on previous experience gathered in preliminary validation studies [12-14]. Two operators were trained on the procedures of the study and performed all evaluations. EL arterialization, aiming at inducing a capillary shunting between arterial and venous territories, was achieved by applying a vasodilation cream (2% nitro-glycerin cream - Pharmacy Department, Corporació Sanitària Parc Taulí. Spain). Arterialization was initially performed during two and a half minutes for the first 20 patients, but was later increased to five minutes for the remaining 35 patients of the study. Reasons for increasing arterialization time were based on the results from the first interim analysis in which high sampling failure was observed. Increasing arterialization time aimed at increasing capillary blood flow at the earlobe in an attempt to improve sampling success rate. Following arterialization, a dedicated collection system (Earlobe Arterialized Blood Collector - EABC®; Microgravity Center/FENG-PUCRS, Porto Alegre, Brazil) was used for collection. Designed as a sterilized, self-contained, small, portable device, the EABC® system allowed anaerobic blood sampling by minimally trained personnel with reduced discomfort to the subject. The EABC® system consists of a plastic shell containing a small blade, a heparinized capillary tube and a sensor cartridge. After proper fixation to the EL, the sensor cartridge receives arterialized blood directly from a small cut in the skin through the capillary tube. Blood was procured from a specific region of the earlobe in a consistent fashion. The sensor cartridge and the capillary tube are visible so that the operator can monitor blood passage as it enters the cartridge until a predefined mark. Samples were analyzed using CG4^+^ gasometrical cartridges and the i-STAT® portable analyzer (Abbot, Abbott Park, IL, USA) [15]. Once patients were included into the study, one blood sample was collected per patient. Inability to obtain a valid blood sample after four collection attempts was considered a sampling failure. Simultaneously, an arterial blood sample was collected from the arterial line and analyzed by the ABL 700® blood gas analyzer (Radiometer, Brønshøj, Denmark) [16]. This system was considered the reference method of measure for this study. Both the i-STAT® and the ABL 700® were calibrated, maintained and updated periodically as indicated by the manufacturer.

### Data collection

Common demographic variables and relevant clinical variables were recorded (Table 1). We considered high heart rate (> 130 beats per minute), low mean arterial pressure (< 65 mmHg), low cardiac output index (< 2.5 L/min/m^2^) measured by thermodilution techniques or by arterial pulse contour analysis, increased lactate levels (> 22 mg/dl) and vasoactive drug treatment, as markers of hemodynamic failure. The main gasometrical variables pH, PO_2_, PCO_2_ and lactate were measured in arterial and EL arterialized capillary samples.

**Table 1:** Demographic and clinical characteristics of study patients

**Patients**	**55**
Male	40 (72.7%)
Age in years	63 (24 to 83)
Arterial hypertension	23 (41.8%)
Diabetes mellitus	19 (34.5%)
Chronic cardiac failure	4 (7.3%)
Severe vasculopathy	4 (7.3%)
Renal insufficiency	5 (9.1%)
Diagnostic at ICU admission	
Severe sepsis^a^	21 (38.1%)
Respiratory failure	7 (12.7%)
Severe trauma	7 (12.7%)
Neurological	6 (10.8%)
Cardiogenic shock	5 (9.1%)
Miscellaneous	9 (16%)
Clinical variables	
PEEP (cmH_2_O)	7.1 (4 to 12)
FiO_2_	0.36 (0.21 to 0.66)
Minute volume (L/min)	9 (5.5 to 19)
APACHE II	18.3 (3 to 34)
Mean arterial pressure (mmHg)	80 (57 to 113)
Cardiac index (L/min/m^2^)	3.2 (1 to 5.9)
Arterial lactate (mg/dl)	18.7 (6 to 71)
Arterial lactate > 22 mg/dl	10 (18%)
Sepsis^a^	32 (58.2%)
Platelets (× 10^3^/dl)	226 (54 to 669)
Prothrombin time (ratio)	1.2 (1 to 1.9)
Prophylactic anticoagulant	40 (72.7%)
Antiplatelet treatment	6 (10.9%)
Vasoactive treatment	36 (65.5%)
NE	30 (83.3%)
NE dose (μg/kg/min)	0.49 (0.02 to 3.02)
Other vasoconstriction treatment	3 (8.2%)
Inotropic treatment	5 (13.8%)
Vasodilation treatment	2 (5.5%)

Data reported as mean (range), except where% is shown in which case values illustrate ‘incidence’. APACHE II, Acute Physiology and Chronic Health Evaluation II score; NE, norepinephrine; PEEP, positive end expiratory pressure.

^a^Following Surviving Sepsis Campaign Guidelines [17].

### Data analysis

Analytical performance and agreement between capillary and arterial samples was evaluated using a combined approach implementing the CCC [18], evaluation of the slope of the regression line equation and the Bland-Altman method [19]. Mean difference (bias), limits of agreement of the mean difference (±2SD) and percentage of error (2SD/mean reference value) were calculated to describe agreement levels of capillary measures with the standard measure method (arterial samples + ABL 700®). Precision of the new system was also evaluated using the coefficient of variation (CV) approach (2SD/mean) [20]. The lower CV, the higher precision of measure and a CV < 10% is desirable in order to enable valid agreement evaluation between sampling/analysis techniques. To this end, up to six samples were obtained from one EL in five critically ill patients (mean = 3.8 (3 to 6) samples per patient). Precision of the reference system (ABL 700®) when analyzing arterial samples is also presented as provided by the producer [16].

The association between study variables and sampling success/failure ratio was evaluated. The Chi-square test was used for qualitative variables, two-tailed Student’s paired *t*-test was used for quantitative variables and Relative Risk (RR) was calculated for interpretation. Any association of hemodynamic instability variables with sampling failure was analyzed individually and in combination using a linear-by-linear association test. Influence of vasoactive drugs, vasopressors and especially norepinephrine (NE) was also evaluated. Threshold drug dose for detecting an effect on sampling outcome was determined by a Receiving Operator Curve (Sampling success/Drug dose) and a Youden test [21]. Finally, a multivariate analysis using logistic regression was performed to find variables independently associated with sampling failure.

ARDS was defined following the established criteria of the Berlin definition [22]. Patients were classified in three groups according to PaO_2_/FiO_2_ ratio (arterial PO_2_ divided by inspired O_2_ fraction): no-ARDS (PaO_2_/FiO_2_ > 300), mild (300 < PaO_2_/FiO_2_ > 200) or moderate - severe (PaO_2_/FiO_2_ < 200). PaO_2_/FiO_2_ ratio was calculated in arterial samples and was compared using a paired *t*-test. Concordance of measures was evaluated using CCC. Kappa coefficient was calculated to evaluate the agreement between the two methods for classifying patients in different groups. Finally, sensitivity and specificity for diagnosing ARDS and moderate - severe ARDS using capillary samples was calculated.

Two-tailed significance threshold was established at *P* < 0.05. Statistical analysis was performed using SPSS version 19 statistical software (International Business Machines. Armonk, NY, USA) and R Cran version 2.15 (R Foundation for Statistical Computing. Vienna. Austria).

## Results

**Figure 1:**
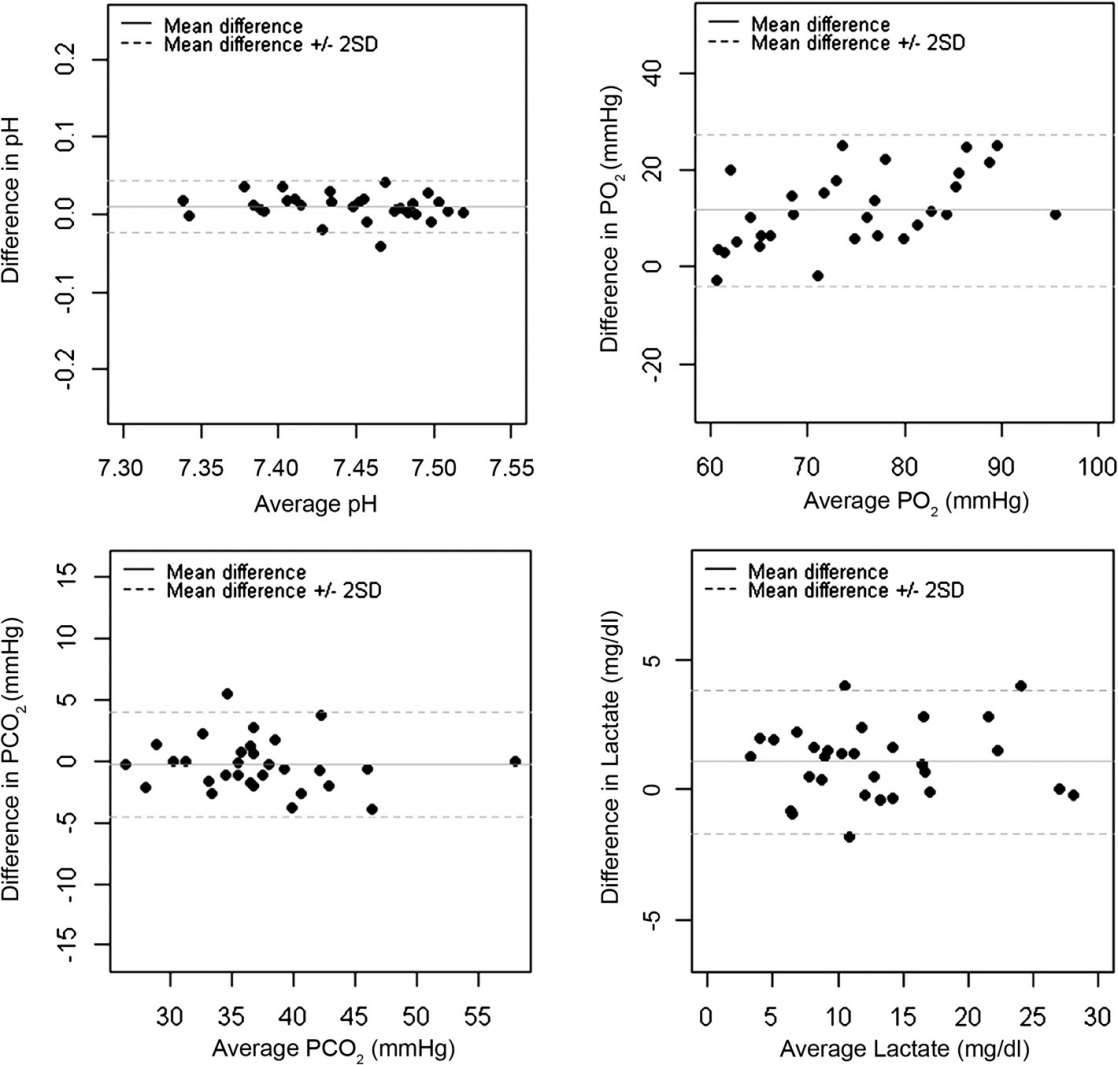
**Differences between capillary and arterial measures.** Bland-Altman plots evaluating pH, PO_2_, PCO_2_ and lactate measure differences between earlobe arterialized capillary blood and arterial blood. X-axis: average arterial measure. Y-axis: mean difference between earlobe arterialized capillary blood samples (EABC®/i-STAT®) and arterial samples (ABL 700®) of each gasometric and lactate measure.

Study patient characteristics were representative of a common ICU patient population of a tertiary hospital (Table 1). EL capillary blood samples were obtained in 56.4% (n = 31) of the cases. Precision levels of EL arterialized capillary samples collected and analyzed using the EABC® + i-STAT® system are presented in Table 2. Precision levels the ABL 700® system when analyzing arterial samples were provided by the producer (Table 2). Arterialized capillary blood showed poor PO_2_ concordance (CCC = 0.45; CI 95% = 0.26 to 0.6) with arterial samples. Capillary measures underestimated arterial values with a statistically significant mean difference of 12 mmHg (*P* < 0.001) (Figure 1). This underestimation increased the higher the patient arterial PO_2_ (slope = 0.54). The accuracy of EL capillary blood measures was low as determined by wide limits of agreement of the mean difference (± 15.5 mmHg) and high percentage of error (19.3%). In a similar manner, PaO_2_/FiO_2_ values were underestimated (mean difference = 34.8; *P* < 0.001; CI 95% = 25.5 to 44) and the magnitude of this underestimation increased the higher the arterial PaO_2_/FiO_2_ (slope = 0.74). Although PaO_2_/FiO_2_ calculation improved concordance (CCC = 0.77; CI 95% = 0.64 to 0.86), measures presented a similar percentage of error (23.8%). On the contrary, a good concordance was observed for PCO_2_ (CCC = 0.94; CI 95% = 0.89 to 0.97) without under or overestimation of values (Figure 1). The range of agreement of the mean difference was ± 4.24 mmHg and the percentage of error was 11.4%. Concordance was also good for pH measures (CCC = 0.93; CI 95% = 0.85 to 0.96) but a statistically significant underestimation was found (mean difference = 0.01 pH units; *P* < 0.001) (Figure 1). Nevertheless, this error was systematic throughout the whole range of values (slope = 0.99). In this case, the range of agreement of the mean difference was ± 0.033 pH units and percentage of error was 0.5%. Lactate measures also showed good concordance (CCC = 0.96; CI 95% = 0.93 to 0.98), however measures of arterialized EL blood were also underestimated (mean difference = 1.07 mg/dl; *P* < 0.001) (Figure 1) but again this error was systematic (slope = 0.97).

**Table 2:** Precision analysis

	**Mean CV% (range)**	
	**Capillary + EABC®/i-STAT®**	**Arterial + ABL 700®**
PO_2_	9.8 (4.2 to 15.8)	4.3 (1.8 to 9.3)
pH	0.3 (0.2 to 0.4)	0.1 (0.08 to 0.1)
PCO_2_	7.7 (1 to 16.1)	3.1 (2 to 5.3)
Lactate	13.8 (3.8 to 21.1)	26.8 (4 to 66.6)^a^

CV, coefficient of variation.

^a^Precision of *in vitro* lactate measures improves with increasing lactate concentrations, therefore mean CV should be interpreted accordingly. At blood lactate concentrations > 2 mmol/L/18 mg/dl, CV decreases below 10%.

Eighteen patients (32.7%) met ARDS diagnostic criteria at the moment of inclusion. Patients were ventilated with a mean PEEP of 8 cm H_2_O (4 to 15) and mean FiO_2_ 0.37 (0.3 to 0.45), and presented a PaO_2_/FiO_2_ of 207 (113 to 299). Sensitivity and specificity for diagnosing ARDS with EL arterialized capillary samples were 100% and 92.3% respectively. Patients were correctly classified into different severity categories (no-ARDS, mild or moderate/severe ARDS; Kappa coefficient = 0.85; *P* < 0.001). In patients with ARDS, sensitivity and specificity for detecting moderate/severe patients (n = 10) were 100% and 75% respectively.

An increased sampling failure rate was evidenced in this study (43.6%) compared to previous validation studies [12-14]. The most prevalent cause of sampling failure was low blood delivery from the EL incision (78.1%). This resulted in insufficient blood flow delivery to the capillary tube, eventually leading to blood flow arrest before reaching the analysis cartridge. Insufficient blood flow was easily detectable by the operator during sampling procedure and was attributed to a reduced capillary flow in the EL. Causes of reduced capillary perfusion were therefore explored. Failure was more frequent in patients older than 65 years (RR = 1.93, *P* = 0.04), in patients treated with doses of NE > 0.22 μg/kg/min (RR = 2.37; *P* = 0.024) and in patients with diabetes mellitus (RR = 1.90 *P* = 0.03). Conversely, sampling success rate was 100% in patients younger than 45 years old (n = 8). Univariate analysis showing no additional study variable was associated with sampling outcome (Table 3). Higher failure rates were observed in patients treated with vasoactive and vasoconstrictor drugs. However, signs of hemodynamic instability were not independently associated with sampling failure. Linear-by-linear analysis showed an increase in sampling failure the more variables were associated although statistical significance was not reached (*P* = 0.146). Increasing vasodilation time from two and a half to five minutes did not affect sampling success rate. Finally, multivariate analysis demonstrated that age was the only variable independently associated with sampling failure (Table 3).

**Table 3:** Factors associated with sampling failure

**Univariate analysis**	**RR**	***P***	**(CI 95%)**
Age > 65 years	1.9	0.04^a^	(1 to 3.7)
Diabetes mellitus	1.9	0.03^a^	(1.1 to 3.4)
Vasoactive treatment	2	0.06	(0.9 to 4.5)
Vasoconstrictor treatment	1.7	0.09	(0.8 to 3.5)
NE > 0.22 μg/kg/min	2.4	0.02^a^	(1 to 5.7)
Sepsis	1.4	0.26	(0.7 to 2.7)
Lactate > 22 mg/dl	1.4	0.38	(0.7 to 3)
MAP (< 65 or > 90 mmHg)	0.8	0.57	(0.4 to 1.6)
**Multivariate analysis**	**OR**	***P***	**(CI 95%)**
Age > 65 years	1.6	0.05^a^	(0.99 to 2.6)
Diabetes mellitus	2.4	0.17	(0.7 to 8.2)
Vasoactive treatment	2.5	0.17	(0.7 to 8.9)

MAP, mean arterial pressure; NE, norepinephrine; OR, odds ratio; RR, relative risk.

^a^Statistically significant.

## Discussion

To our knowledge this validation study is the first to evaluate analytical performance, diagnostic capabilities and applicability of the EL arterialized capillary blood gas analysis technique in a cohort of critically ill patients with varied medical conditions and severity. Arterialized capillary blood gas measurement from the EL has been extensively evaluated in various physiological and pathological situations. Nevertheless, contradictory results as to its accuracy have been reported, especially for PO_2_ measures [2-8]. In a recent meta-analysis authors reported that, although agreement was not high (residual standard error of 6 mmHg in PO_2_ measures), earlobe capillary estimations could be used for clinical management since they followed arterial values [9]. The main sources of variability were attributed to different collection techniques and procedures. Our results have evidenced a high level of precision of capillary gasometrical analysis when a dedicated collector and procedures are used to homogenize the collection technique (CV < 10% in all gasometrical variables - Table 2). However, results from the present analysis demonstrate that arterialized capillary blood is not a good estimator of arterial PO_2_ values in critically ill patients due to poor concordance, underestimation of values, wide limits of agreement and elevated error in the measure. As previously reported, the higher the arterial PO_2_ the higher the discrepancy between capillary and arterial blood measures, which could be explained by poor arterialization and excessive venous blood admixture [9]. Nevertheless, our findings remained unchanged in spite of implementing a means for augmenting EL arterio-venous capillary shunt (vasodilatation cream and extended arterialization times). Adjusting for FiO_2_ values (PaO_2_/FiO_2_) only improved concordance of measures, but underestimation and significant percentage of error were still present. This could be explained by a ‘normalization’ phenomenon when adjusting for FiO_2_ values in patients with and without hypoxemia. Conversely, pH, PCO_2_ and lactate measures were in good agreement with arterial samples over a wide range of values, provided an appropriate offset was applied in some cases. Since arterio-venous PCO_2_ differences are smaller, venous blood admixture may have a reduced impact upon measures. Results showed lactate measure precision was slightly above desirable levels (CV = 13.8%) , however this could be attributable to different analytical sensitivity of the equipment used at different blood lactate levels. Precision of both the i-STAT® and the ABL 700® systems measured *in vitro* improves significantly at lactate levels > 18 mg/dl/2 mmol/L (reported CV = 10% and 6.6% respectively).

We were able to find one preceding study evaluating the accuracy of EL capillary blood gas analysis for predicting arterial values in the ICU environment [23], in which PO_2_ agreement evaluation results were similar to ours while PCO_2_ and pH measures showed good correlation with the standard and were considered accurate enough. Such disparity between the two studies may be due to differences in population characteristics. In the study of Honarmand and Safavi strict inclusion/exclusion criteria reduced representativeness of its population by excluding patients with severe hemodynamic alterations, high FiO_2_ or PEEP values, severe sepsis and multi-organ failure [23]. Conversely, we included a broader spectrum of patients to resemble a real population of critically ill patients.

In patients admitted to the ICU, adequate management often requires accurate gasometric evaluation (that is for ventilator adjustment). Although precision of capillary samples was high, they did not yield a sufficient level of agreement with the reference method in PO_2_ measures and presented excessive dispersion of measures for pH and PCO_2_ for this purpose (± 0.033 units for pH and ± 4.24 mmHg for PCO_2_). Therefore, they would not be recommended for its use in stable critical patients admitted to the ICU and should not replace arterial measurements. On the other hand, when extreme gasometric values are present in the acute phase of many critical conditions, such as in the onset of ARDS, this level of inaccuracy becomes irrelevant. Accordingly, this technique was capable of accurately detecting extreme gasometrical values, and permitted diagnosis and classification of patients presenting with severe respiratory failure and ARDS. In light of the present results, EL capillary blood gas analysis should be considered for its use for the initial diagnosis and emergency management of critically ill patients when less accurate but easier and faster techniques may be preferred. Furthermore, in situations where gasometric evaluation capabilities are not easily available such as in pre-hospital critical care, advanced medical transport, and remote environments medicine this technique can provide relevant information to improve patient diagnosis and management with potential benefits for patient outcome.

A second important finding of this study was a high EL capillary sampling failure rate. This problem had not been evidenced in preliminary validation studies [12-14] and was considered to be independent from the new collecting system. The two interim analyses, undertaken after the inclusion of the 15^th^ and 45^th^ patients, permitted the research team to survey sampling success evolution. High sampling failure ratios were evidenced early in the present study and were clearly associated with insufficient blood flow delivery to the collecting system. This was attributed to a reduced capillary blood flow in the EL. Thereafter, it was decided to increase arterialization time in an attempt to increase vasodilation and augment earlobe subcutaneous capillary blood flow. Nevertheless, extended arterialization times were not able to compensate for insufficient blood flow, and sampling failure rates remained unchanged throughout the study. Upon study finalization, data analysis showed that patient age (> 65 years) was an independent factor associated with sampling failure. Aging is responsible for vascular function impairment [24] and increased capillary rigidity, which may cause limited capillary blood flow and blood delivery to the collector. The impact of age became evident when patients aged < 45 years were analyzed, in which case sampling success rate reached 100%. Indeed, if proper patient selection is made a significant number of patients could benefit from this technique. In our polyvalent ICU, around 48% of patients admitted are < 65 years, representing more than 600 potential users of this technique per year. Finally, vasoactive drugs, moderate NE doses (> 0.22 μg/kg/min) and the cumulative effect of hemodynamic failure variables were also factors with potential influence upon sampling success; however, data did not reach statistical significance. Interestingly, signs of tissue hypoxia/hypoperfusion such as elevated blood lactate or cutaneous signs of insufficient perfusion, and conditions known to cause microvascular dysfunction such as sepsis [25] were not associated with increased sampling failure ratios. This finding questions the impact of low tissue perfusion associated with hemodynamic instability as an influencing factor in sampling success; however the present study was not specifically designed to address this issue.

This study has a number of limitations that must be taken into consideration. Firstly, the reduced number of successful blood collections may be considered as a limitation and could reduce validity of presented results. Furthermore, the reduced number of patients presenting with ARDS limited, and may have biased, the evaluation of the diagnostic capability of capillary analyses of highly altered gasometrical values. However, the study reached the required statistical power to demonstrate concordance of the methods being analyzed by the statistical approach selected. Secondly, although sampling failure was attributed to patient characteristics, design problems with the new collecting system could have influenced results. Nevertheless, the EABC® system and the i-STAT® portable analyzer have already been used together satisfactorily in several validation studies [12,14] where none of the present problems could be detected. Thirdly, we decided to include a broader spectrum of critically ill patients in order to improve representativeness of the data. However, this complicated interpretation of results by adding a number of uncontrolled variables, which could have had an impact on the results. Finally, the utilization of two different analyzers creates a possible problem of comparability. The ABL 700® system was the best gold-standard system available, presenting adequate accuracy and precision levels and allowing the research team to be consistent with current analytical methodology used in our ICU. On the other hand, the i-STAT® portable analyzer was the only system that can be used with the collection system used in this study (EABC®). Notwithstanding these limitations, concordance levels achieved for pH, CO_2_ and lactate values reported across collection and analysis techniques were supportive of the fact that the type of analyzer used did not influence results significantly.

## Conclusion

This validation study suggests that EL arterialized capillary blood gas analysis is precise, capable of detecting extreme gasometric values in selected critically ill patients, and could permit diagnosis and classification of patients presenting with ARDS. Although it may present some accuracy and applicability problems for accurate management of patients already admitted to the ICU, it could be used for initial diagnosis and emergency management of critically ill patients. In medical scenarios where less accurate but easier and faster techniques for gasometric evaluation may be beneficial, or in situations where no analytical capabilities are easily available, EL arterialized capillary blood gas analysis at point-of-care with dedicated sampling equipment and procedures can offer significant advantages over traditional methods. In light of these preliminary results, further investigation should be considered in other critical care arenas, such as in emergency medicine, advanced medical transport and pre-hospital critical care where this technique could offer decisive information with potential influence in patient outcome.

## Abbreviations

APACHE II: Acute Physiology and Chronic Health Evaluation II score; ARDS: Acute Respiratory Distress Syndrome; CCC: Concordance Correlation Coefficient; CV: Coefficient of Variation; EABC®: Earlobe Arterialized Blood Collector®; EL: earlobe; NE: norepinephrine; OR: odds ratio; PEEP: positive end expiratory pressure; RR: Relative Risk.

## Competing interests

The authors declare that they have no competing interests.

## Authors’ contributions

SV contributed to the study conception and design, collected and analyzed data, interpreted results and was responsible for the drafting, review and final approval of the manuscript. JM contributed to the study conception and design, collected data and interpreted results, and participated in the manuscript drafting, review and final approval. GG and GG both contributed to patient screening and inclusion, collected data and participated in the manuscript review and final approval. JCO contributed to study conception and design, analyzed data, interpreted results, and reviewed and approved the final manuscript. AF contributed to study conception and design, interpreted results, and reviewed and approved the final manuscript. SE contributed to study conception and design, interpreted results, and reviewed and approved the final manuscript. TR contributed to study conception and design, interpreted results, and reviewed and approved the final manuscript. AA contributed to study conception and design, interpreted results, and reviewed and approved the final manuscript.
